# Molecular epidemiology of rotavirus causing diarrhea among under-five children after the introduction of rotavirus vaccines, Rotavac and Rotasiil, into the national immunization program of India

**DOI:** 10.1186/s12985-026-03126-0

**Published:** 2026-03-14

**Authors:** Tintu Varghese, Anupama Machathi, Shainey Alokit Khakha, Nayana P. Nair, Reddy N. Samarasimha, Varsha Sudhir Chaudhary, Namrata Kharat, Prasanna Samuel, Sidhartha Giri, Venkata Raghava Mohan, Gagandeep Kang

**Affiliations:** 1 The Wellcome Trust Research Laboratory, Christian Medical College, Vellore, India; 2 Indian Council of Medical Research - National Institute of Nutrition, Hyderabad, India; 3 Department of Biostatistics, Christian Medical College, Vellore , India; 4 Indian Council of Medical Research - Regional Medical Research Centre, Bhubaneswar, India; 5 Department of Community Medicine, Christian Medical College, Vellore, India

**Keywords:** Genotyping, Rotavirus strains, Diarrheal surveillance, Vaccine impact, Rotavirus vaccines, Gastroenteritis

## Abstract

**Background:**

India was among the first Southeast Asian countries to introduce rotavirus vaccines (Rotavac and Rotasiil) into its Universal Immunization Programme (UIP). Both showed efficacy in trials and protection against multiple genotypes, though concerns remain about cross-protection and strain selection.

**Methods:**

We leveraged multicentric surveillance studies to assess the impact of vaccination on circulating rotavirus strains. From 2016 to 2023, hospital-based diarrheal surveillance enrolled children under five hospitalized with acute watery diarrhea. Stool samples were tested by rotavirus enzyme immunoassay (EIA), and positives were genotyped.

**Results:**

Among 27,862 samples, 6755 (24·2%) were rotavirus EIA positive, with the highest positivity in eastern and northeastern regions. Positivity declined from 31·2% to 17·5% over the study period, compared to 37% before vaccine rollout. Genotype distribution was broadly consistent across regions, dominated by G3P[8] (44·7%), G1P[8] (14·7%), G2P[4] (14·1%), and G1P[6] (4·5%). Mixed infections occurred in 13·9%. G12P[8] was frequent in the northeast. Post-vaccine introduction, G3P[8] and G2P[4] increased while G1P[8] declined. Genotype patterns differed by vaccine type: G2P[4] was more common at Rotasiil sites (30·9%) than Rotavac sites (10·2%), while mixed infections were lower at Rotasiil sites (7·8%) compared to Rotavac sites (15·3%).

**Conclusions:**

Rotavirus vaccination significantly reduced disease burden among under-five children in India. G3P[8] remained consistently dominant across regions, vaccine types, and years. Regional and vaccine-specific differences were evident, but trends suggest natural viral evolution may outweigh vaccine-driven selection. Sustained surveillance is essential to detect emerging strains and ensure long-term vaccine effectiveness.

**Supplementary Information:**

The online version contains supplementary material available at 10.1186/s12985-026-03126-0.

## Background

Group A Rotavirus is a leading cause of severe diarrheal illnesses among children worldwide. Rotavirus caused 122,000 to 215,000 deaths annually in children under five years of age as per the World Health Organization (WHO) (2013–2017) estimates, with India accounting for 16% of the global mortality burden [1]. In countries without rotavirus vaccines, rotavirus causes 40% of childhood diarrheal hospitalizations [2]. Before the introduction of the rotavirus vaccine in India, approximately one out of every 31 children was hospitalized for rotavirus-related diarrhea by the a of five [3] In 2006, two rotavirus vaccines - Rotarix and RotaTeq - received global licensure, and in 2009, the WHO recommended rotavirus vaccines inclusion in the national immunization programs [4] Between 2006 and 2019, these vaccines were estimated to have saved around 140,000 lives globally, with countries observing a 59% reduction in rotavirus-related hospitalizations and a 36% decline in severe diarrheal deaths [5].

On 26 March 2016, India became the first nation in the WHO South-East Asia Region to incorporate the rotavirus vaccine into its Universal Immunization Program [6].24/11/2025 08:49:00 The monovalent oral vaccine Rotavac (Bharat Biotech), based on the G9P[11] human-bovine reassortant 116E strain, was initially launched in four states and gradually expanded in a phased manner. In 2018, the pentavalent oral vaccine Rotasiil (Serum Institute of India), containing G1, G2, G3, G4, and G9 antigens, was also added to the UIP to achieve nationwide coverage by 2019 [7]. The distinct antigenic composition of Rotavac and Rotasiil, in contrast to globally licensed vaccines such as Rotarix (monovalent G1P[8]) and RotaTeq (pentavalent G1, G2, G3, G4, P[8]), raises important questions regarding their respective effects on circulating rotavirus genotype diversity and evolutionary dynamics in India.

Rotaviruses are classified based on the outer capsid proteins VP7 (G type) and VP4 (P type), with genotypes G1P[8], G2P[4], G3P[8], G4P[8] and G9P[8] historically accounting for the majority of global disease burden [8]. The segmented nature of the rotavirus genome facilitates frequent reassortment events, contributing to extensive genetic diversity and enabling the emergence of novel or uncommon strains. Co-infection with multiple rotavirus strains (mixed infections) further enhances their potential for reassortment and genetic diversification. Since the introduction of rotavirus vaccines, several countries have reported shifts in the genotype distribution. For example, in countries predominantly using the Rotarix vaccine, such as Brazil and Australia, G2P[4] strains have transiently increased in prevalence, whereas increases in G12P[8] strains have been observed in countries using the pentavalent RotaTeq vaccine [9, 10] Additionally, longitudinal surveillance in settings such as Belgium and Australia has documented an increased proportion of equine-like reassortant G3P[8] strains following vaccine implementation, raising the possibility of vaccine-induced selective pressures [11, 12]. Nevertheless, genotype patterns exhibit temporal variability, with multiple strains co-circulating annually, independently of vaccine introduction [13].

Despite the demonstrated benefits of rotavirus vaccines, limited evidence exists on how the two Indian vaccines impact the genetic landscape of circulating rotavirus strains. Given the distinct vaccine compositions and the wide heterogeneity of rotavirus genotypes observed in India, the long-term effects of vaccination on strain dynamics remain unclear. We hypothesize that the use of a monovalent rotavirus vaccine is associated with the emergence of non-vaccine rotavirus strains due to selective immune pressure, whereas a multivalent vaccine provides broader cross-protection, thereby reducing the prevalence and diversity of emerging strains. This study presents findings from eight years of post-vaccine surveillance (2016–2023) across multiple regions in India. The primary objectives of this study were to describe the distribution of rotavirus genotypes in India following vaccine introduction and to compare genotype trends across regions and between sites using Rotavac and Rotasiil.

## Methods

### Study sites

Multicenter hospital-based diarrheal surveillance was conducted in India from 02 April 2016 to 30 December 2023 following the introduction of the rotavirus vaccines, Rotasiil and Rotavac to assess the vaccines’ impact. These studies adhered to similar protocols for case enrolment, data collection, and laboratory testing [14] The Rotavac Impact Assessment Study was conducted across 31 sentinel sites in nine states from 2016, while the Rotasiil Impact Assessment Study was conducted across 27 sites in five states from 2019 in India [7]. Details of the sentinel sites, vaccine introduction date and duration of surveillance are provided in *S1 Table 1*. Data collected from diarrheal surveillance were analysed to understand the diversity and distribution of rotavirus genotypes after the upscaling of rotavirus vaccines in India.

### Enrolment and data collection

Children < 60 months of age admitted with acute gastroenteritis for ≥ 6 h were enrolled in the study. Patients referred from other hospitals after 24 h of admission were excluded. Clinical, sociodemographic, and vaccination details were collected at the time of enrolment.

### Sample collection and transport

Stool samples were collected within 48 h of admission and transported to the sentinel site laboratory within 2 h of collection and stored at -20 °C or (-80 °C whenever possible) in the sentinel sites until shipment. These samples were shipped monthly to CMC Vellore, which is the study coordinating and testing center.

### Laboratory procedure

Stool samples were screened for rotavirus VP6 antigen using WHO-recommended enzyme immunoassay (EIA) kits (Premier Rota clone, Meridian Bioscience). EIA-positive samples were genotyped to identify VP7 (G type) and VP4 (P type) genes using reverse transcription polymerase chain reaction (RT-PCR) assays, following published protocols [15–17]. Viral RNA was extracted from a 20% stool suspension, followed by reverse transcription to generate complementary DNA (cDNA) using random primers (Invitrogen) and Moloney murine reverse transcriptase enzyme (Superscript II MMLV-RT, Invitrogen). The cDNA was then used in hemi-nested multiplex RT-PCR assays to identify the G (G1, G2, G3, G4, G8, G9, G10, and G12) and P (P [4], P [6], P [8], P [9], P [10], P[11] ) types using published oligonucleotide primers. Untyped samples were further tested using VP6 conventional PCR to rule out false-positive EIA results. Sanger sequencing was used to confirm unusual genotypes such as G1P[4], G2P[6], G2P[8], G3P[4], G3P[6], G4P[6], G10P[11], G12[P4], and G12P[11] [17].

### Statistical analysis

Clinical and laboratory data were analysed to evaluate the proportion of rotavirus-associated diarrhea, genotype diversity, and temporal and regional variation in rotavirus genotypes across various geographical zones (north, south, east, west, central and northeast) from 2016 to 2023. All statistical analyses were performed using the R software (Version 4.2.1; R Core Team, 2022).

### Ethics statement

Ethical approval was obtained from the Institutional Review Board of Christian Medical College, Vellore (IRB No: 14008). Participants were enrolled into the study after obtaining written informed consent from the parent/guardian.

## Results

### Decline in rotavirus positivity

**Fig. 1:**
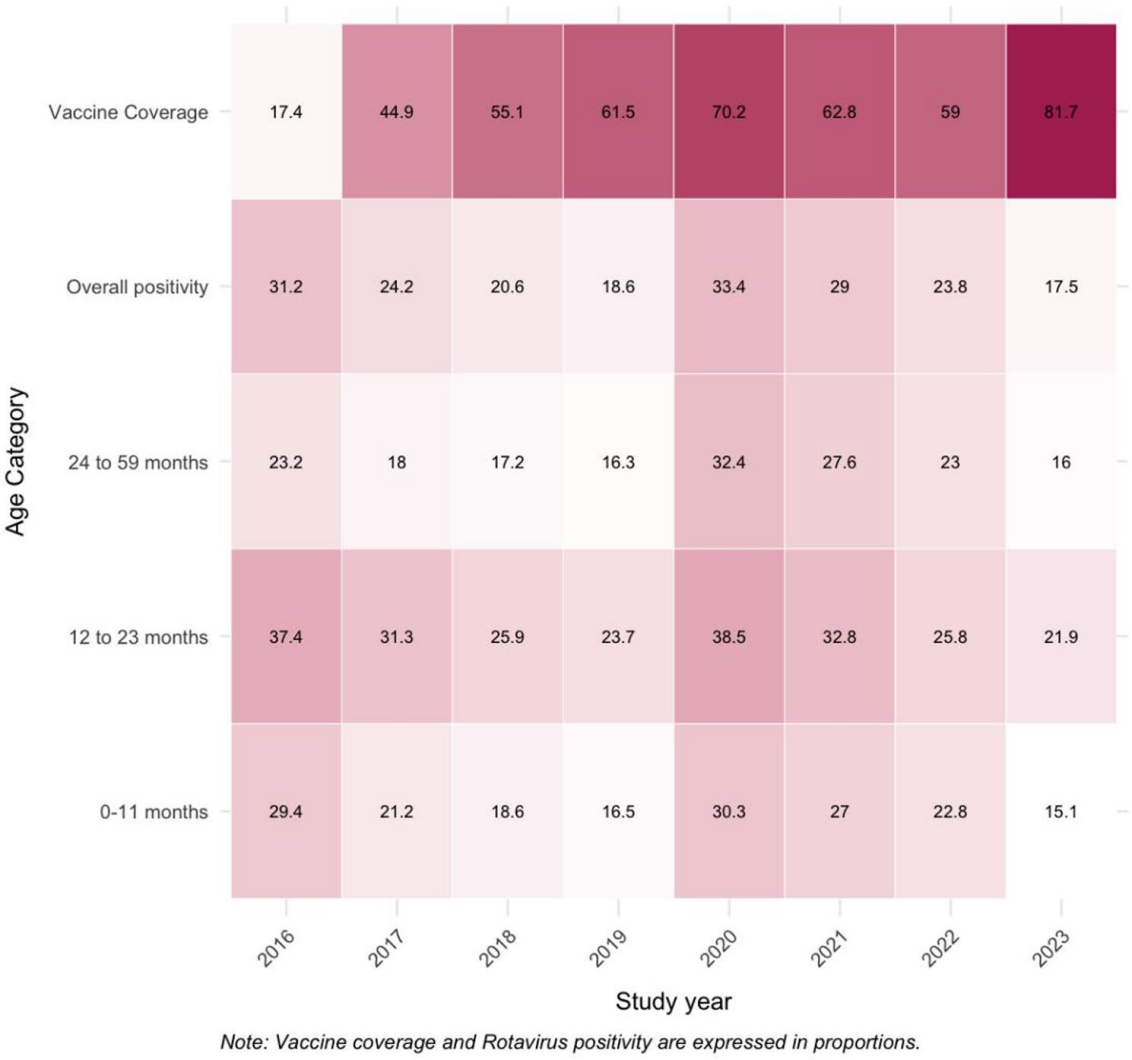
Trends in rotavirus positivity among Indian children in the post-vaccine era

During the study period, 30,044 children were enrolled in the study, of which 27,862 (92·3%) stool samples were available for rotavirus EIA testing. Of these, 6755 (24·2%) samples were positive for rotavirus EIA. Rotavirus diarrhea was found throughout the year in all geographical regions in India, although the positivity rate was higher in northeastern (36·7%) and eastern (36·1%) India than in the rest of India (west − 20·9%, south-19·1%, central-18.9%, north-18·8%) (*S1 Table 2*). Rotavirus positivity demonstrated a steady decline over the study period, with the exception of 2020, when a transient increase was observed. A reduction in positivity was evident across all age groups (Fig. 1), with the most pronounced decline among infants (0-11months of age) (29·4% in 2016 to 15·1% in 2023; 48·6% reduction). Toddlers showed a 41·4% decrease, preschool-aged children a 31·0% decrease, and the overall positivity declined by 43·9%. The data show a clear inverse relationship between increasing vaccine coverage and declining rotavirus positivity from 2016 to 2019. The temporary deviation after 2019 likely reflects COVID-19–related disruptions to immunization and surveillance, after which the declining trend resumes, consistent with a sustained population-level impact of vaccination.

### Rotavirus genotype distribution and trends over time

Among the rotavirus EIA-positive samples, 6692 (99·1%) were genotyped. Overall, the most common genotype was G3P[8] (44·7%) followed by G1P[8] (14·7%), G2P[4] (14·1%), G1P[6] (4·5%), G9P[4] (2·9%) and G12P[8] (1·6%). Some uncommon strains, including G2P[8] (*n* = 3), G3P[4] (*n* = 7), G3P[6] (*n* = 14), and G12P[11] (*n* = 11), were occasionally identified during the study period constituting up to 1·6% of strains. Of the samples, 64 (1·0%) were only partially typed, and 4 (0·1%) remained untyped. Throughout the study, mixed infections accounted for 13·9% of the cases.

**Fig. 2:**
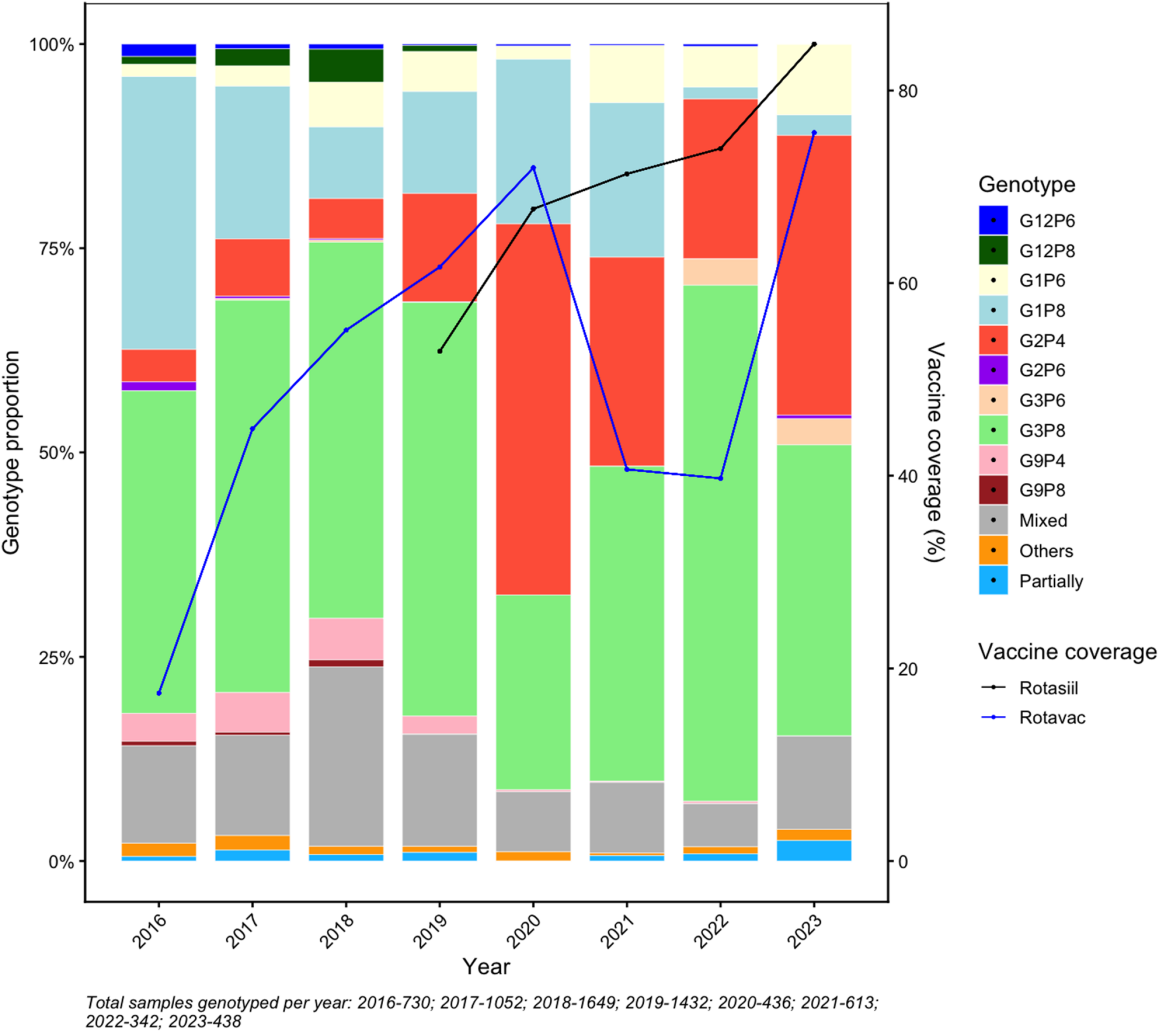
Trends in rotavirus genotypes circulating among under five Indian children (2016–2023)

The distribution of rotavirus genotypes varied throughout the study period (2016–2023) (Fig. 2). G1P[8] was predominant in 2016 and 2017 but declined thereafter, becoming minimally detected by 2022. The G3P[8] was the dominant genotype throughout the study period (except in 2020). A notable shift occurred in 2020, where G2P[4] became the predominant genotype, surpassing G1P[8] and G3P[8] and has been in circulation in a higher proportion than in previous years. This coincides with the period of COVID-19–related disruption to immunization services and the early phase of Rotasiil scale-up, during which Rotavac coverage declined. Importantly, despite continued increases in Rotasiil coverage after 2020, the proportion of G2P[4] did not show a sustained increasing trend, suggesting that this transient dominance is more consistent with natural temporal fluctuation during a period of programmatic transition rather than a simple vaccine-driven replacement.

### Variation in genotype patterns by region

**Fig. 3:**
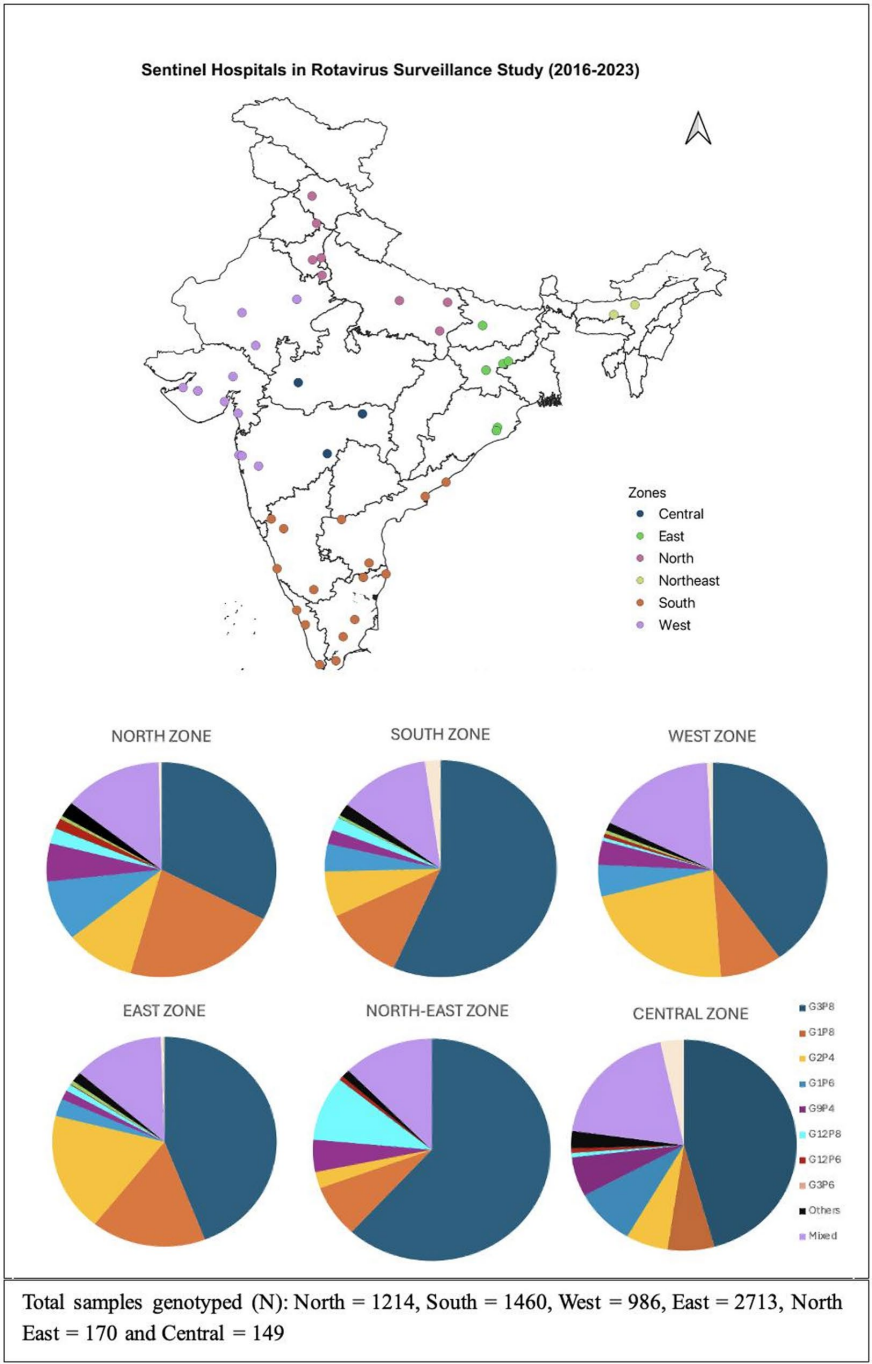
Distribution of rotavirus genotypes in the various regions of India

The distribution patterns of various genotypes across regions are shown in Fig. 3. The genotype distribution was similar across all regions of India, with G3P[8] followed by G1P[8], except in the eastern and western regions, where G2P[4] was more common than G1P[8]. G12 P[8] was the second most common strain in the northeast.

### Genotype distribution by vaccine type across india

**Fig. 4:**
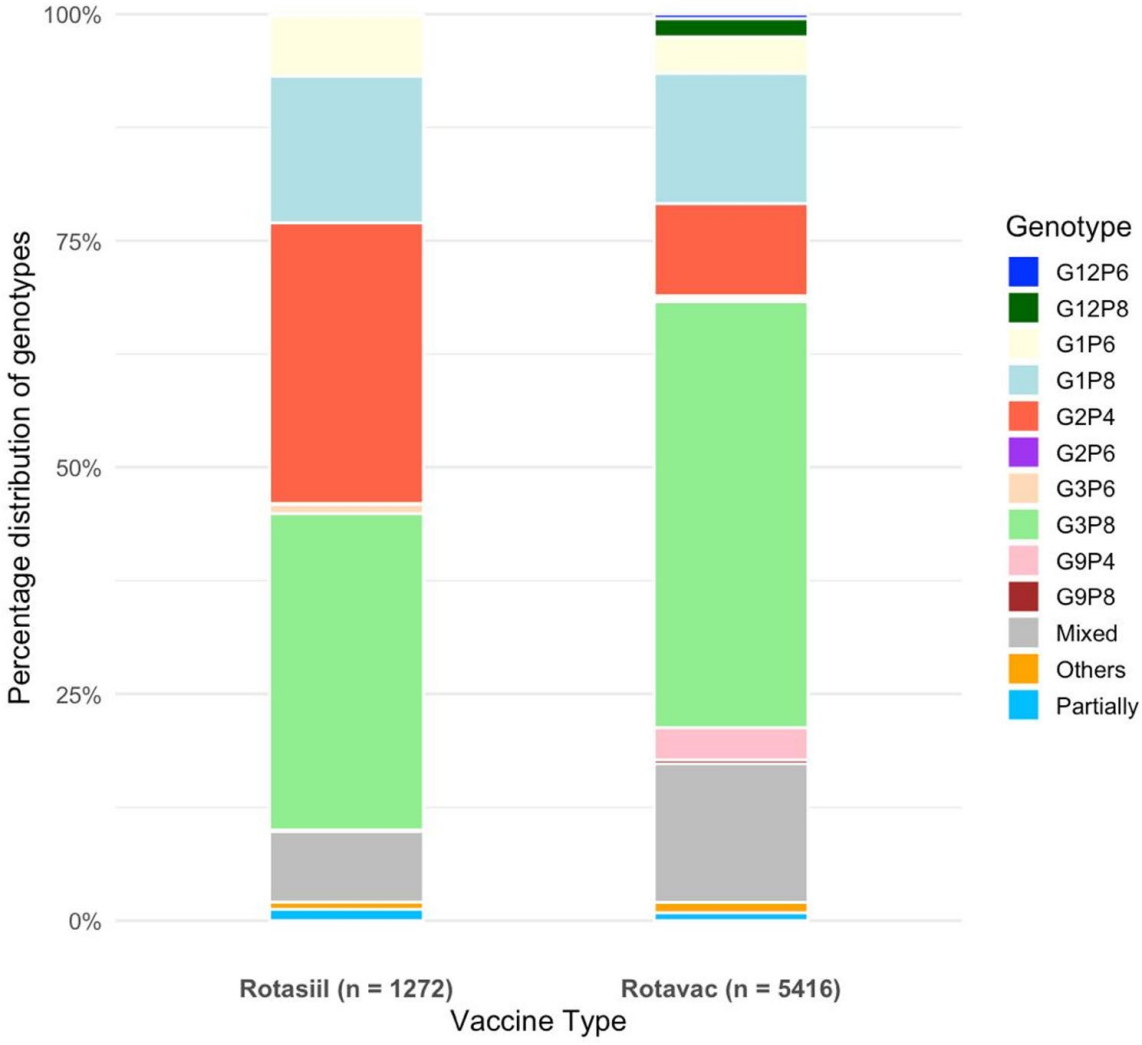
Rotavirus genotypes based on vaccine type: Rotavac versus Rotasiil (2016–2023)

To explore the influence of different vaccines on circulating rotavirus strains, the genotype distribution was compared across Indian states using Rotavac or Rotasiil. Overall, the distribution patterns mirrored national trends, with G3P[8], G1P[8], and G2P[4] being the most common circulating strains (Fig. 4). Across both vaccine groups, G3P[8] emerged as the predominant genotype, accounting for 47·0% of cases in Rotavac sites and 34·9% in Rotasiil sites. In Rotavac-using states, G1P[8] (14·4%) was more prevalent than G2P[4] (10·2%), and mixed infections were observed in up to 15·3% of samples. In contrast, Rotasiil sites reported a higher proportion of G2P[4] (30·9%) than G1P[8] (16·2%), with mixed infections of up to 7·8%.

## Discussion

The overall rotavirus positivity was 24·2% among 27,862 samples tested during the eight years following vaccine introduction - a marked decline from the 37% reported in a multicentre study prior to rotavirus vaccine rollout [18]. Our findings align with previous reports that have documented a decline in rotavirus-associated hospitalizations after vaccine introduction in India [19, 20].

Pre-vaccine surveillance in India identified geographic differences in rotavirus positivity, with higher rates in the east (40·3%) than in the south (35·7%) and west (34·9%) [18]. Our post-vaccine data showed a similar trend, with higher positivity in the east (36·1%) relative to the south (19·1%) and west (21·0%). Despite persistent regional variation, a clear decline in positivity across all regions highlights the broad impact of immunization in reducing the disease burden nationwide.

A reduction in rotavirus-related diarrhea was observed across all age groups under five years, with the most notable decrease in infants - consistent with expectations, as infants were the primary targets of vaccination. Previous evidence also suggests that rotavirus vaccines provide indirect protection, contributing to reduced diarrhea incidence in older children [21, 22].

Although the overall decline in rotavirus positivity was sustained over the years following the vaccine introduction, a temporary increase was observed during the COVID-19 pandemic. This may be attributed to disruptions in routine immunization services or shifts in healthcare-seeking behaviour, where only more severe cases reached hospitals. Similar trends have been reported in Bangladesh and Turkey [23, 24], while other studies have documented a decline in rotavirus infections, largely attributed to public health measures such as social distancing and mask use that reduced pathogen transmission [25, 26] These observations highlight the complex interplay among public health measures, pathogen transmission, and potential changes in healthcare access during pandemics.

In our study, nearly 75% of rotavirus infections were attributed to three genotypes; G3P[8] (44·7%), G1P[8] (14·7%), and G2P[4] (14·1%). This pattern was consistent across most regions of India, except in the northeast, where G12P[8] was the second most common strain after G3P[8]. G12 strains, although less common nationally, have historically circulated in North India, as supported by earlier studies [27, 28] Several other post-vaccine studies from India have identified G3P[8] as the most common strain [29, 30]. In a pre-vaccine study conducted in India (2005–2016), G1P[8] (38·7%) and G2P[4] (12·3%) constituted over half of the rotavirus positive samples, indicating that there have been no significant shifts in strain prevalence since the introduction of the vaccine, apart from an increase in G3P[8] [18, 19].

We observed a steady rise in the prevalence of G3P[8] and a decline in G1P[8] during the surveillance period. These genotype trends were already evident in India before the national introduction of rotavirus vaccines [19]. Global rotavirus surveillance network data (2014–2018) have reported the frequent detection of G3P[8] following vaccine introduction [31]. However, this strain has also been reported in countries without vaccination programs, suggesting that its emergence is likely driven by natural evolutionary and ecological dynamics rather than vaccine pressure. For example, in Southeast Asia, G3P[8] has predominated in both vaccinated (e.g., Philippines) and unvaccinated (e.g., Thailand, Myanmar) settings [31–33]. The decline in G1P[8] has similarly been documented worldwide and within India since around 2014, even before the introduction of rotavirus vaccines into India’s UIP [18, 31, 33]. These findings suggest that post-vaccine genotype changes are influenced by natural evolutionary and ecological dynamics, rather than vaccine pressure.

Our study identified a relatively high proportion of mixed rotavirus infections (13·9%), compared to the 6·7% reported in similar Indian settings from 2005 to 2016 [18]. Mixed infections are important for monitoring, as they can result in reassortment events and the emergence of novel genotypes with altered antigenic properties, thereby posing challenges for sustained vaccine effectiveness. We also detected several uncommon genotypes, including G1P[4], G2P[6], G2P[8], G3P[4], G3P[6], G10P[11], G12P[4], and G12P[11], consistent with previous reports from India indicating continued circulation of diverse strains post-vaccine introduction [18].

Comparative analysis of genotype distribution across Indian states using either Rotavac or Rotasiil offers valuable insight into potential vaccine-related influences on circulating rotavirus strains. Although the overall patterns generally followed national trends, some differences were observed between the two vaccine groups, which should be interpreted with caution. Diarrheal surveillance in sites administering Rotavac began in 2016, whereas sites using Rotasiil were included only from 2019 onward. As a result, the two groups reflect different phases of strain circulation, making direct vaccine-specific comparisons challenging. Across both groups, G3P[8] emerged as the most common genotype, accounting for nearly half of the cases in Rotavac sites and over a third in Rotasiil sites. In states using Rotavac, G1P[8] (14.4%) and G2P[4] (10.2%) were circulated in similar proportions, whereas in Rotasiil-using states, G2P[4] was more commonly detected than G1P[8] (30·9% vs. 16·2%). This aligns with the findings of Phase III clinical trials of both these vaccines conducted in India and Niger [34, 35]. Higher rates of mixed infections were observed at Rotavac sites (15·3%) than at Rotasiil sites (7·8%). This variation is interesting, especially given the differing compositions of the two vaccines - Rotavac being monovalent (G9P[11]) and Rotasiil being pentavalent (including G1–G4 and G9).

Similar patterns have been documented globally for other vaccine types, including Rotarix and RotaTeq and are increasingly understood to reflect natural strain dynamics rather than direct vaccine pressure. For example, the emergence of G2P[4] following the Rotarix introduction in Australia and Brazil was initially thought to be vaccine-driven [10, 36], However, historical data show that G2P[4] re-emerges cyclically every 3–5 years, including in non-vaccinated populations, suggesting that strain replacement can occur independently of vaccine composition [37]. Likewise, G3P[8], which was the predominant genotype at both Rotavac and Rotasiil sites in this study, has been reported in RotaTeq/Rotarix-using countries such as Australia and the U.S. [10, 38], as well as in unvaccinated settings such as Vietnam and Japan [39, 40].

The emergence of equine-like G3P[8] strains has been documented in several countries following the introduction of Rotarix and RotaTeq, prompting a discussion on the potential role of vaccine-driven immune pressure in the selection of these reassortant strains [12, 41, 42]. In our study, G3P[8] was the predominant genotype, however, whole genome sequencing (WGS) data are currently unavailable to determine whether these strains share characteristic with the globally reported equine-like variants. (Sanger sequencing of these samples is underway, and comprehensive results will be presented in a separate manuscript). This information is particularly relevant in the Indian context, where different vaccines such as Rotavac and Rotasiil - have been used in the national immunization program.

In summary, while vaccine type may influence genotype distribution to some extent, the observed patterns are more consistently aligned with natural epidemiologic fluctuations and the timing of surveillance activities. This reinforces the importance of sustained, multi-site molecular surveillance to capture evolving genotype trends, and identify uncommon or emerging strains.

A key strength of our study is the standardized case recruitment across multiple sites in India, ensuring consistent data collection and minimizing site-level variability. All stool samples were tested in a single, centralized reference laboratory following the WHO rotavirus surveillance standards, enhancing the reliability and comparability of genotype data across regions and over time. A limitation is that not all sites participated throughout the entire study period, which may have affected the temporal trend analysis and limited the generalizability of the findings for certain timeframes. Nonetheless, the multi-site design provides a comprehensive overview of rotavirus genotype circulation across diverse geographic areas and vaccine-use settings in India.

## Conclusion

This eight-year, multi-site surveillance study demonstrated the substantial and sustained impact of rotavirus vaccination in India, as evidenced by a marked decline in rotavirus positivity and disease burden among children under five across diverse geographic regions. Changes in genotype distribution, including the rise in G3P[8] and G2P[4] and the decline in G1P[8], were observed over time. However, similar trends in vaccinated and unvaccinated settings globally suggest that such shifts are more likely due to natural evolutionary dynamics rather than vaccine-driven selective pressures. Nevertheless, the ongoing circulation of diverse and mixed rotavirus strains highlights the need for continued molecular surveillance. The predominance of G3P[8] in particular warrants further investigation through whole genome sequencing to identify potential equine-like variants, especially considering the different vaccine formulations in use in India. Strengthening genomic surveillance augmented with whole genome sequencing will be critical for monitoring viral evolution, informing vaccine effectiveness, and guiding future vaccine policies.

## Supplementary Information

Below is the link to the electronic supplementary material.

Supplementary Material 1.

## Data Availability

De-identified data will be made available upon reasonable request to the corresponding author. Access will be granted to investigators whose proposed use of the data has been reviewed and approved by an independent review committee established for this purpose. Requests should be addressed to tintu.varghese@cmcvellore.ac.in.
