# Supplementary material for: Molecular epidemiology of rotavirus causing diarrhea among under-five children after the introduction of rotavirus vaccines, Rotavac and Rotasiil, into the national immunization program of India
Source: Virol J. 2026 Mar 14;23:104. doi: 10.1186/s12985-026-03126-0 (PMC13101206; doi:10.1186/s12985-026-03126-0)

**Molecular epidemiology of rotavirus causing diarrhea among under-five children after the introduction of rotavirus vaccines, Rotavac and Rotasiil, into the national immunization program of India**

**Table of contents**

Supplementary Table 1. Sentinel sites and duration of diarrheal surveillance in India (2016 - 2023) - Page 2

Supplementary Table. 2 Distribution of rotavirus positive samples by zone, based on EIA testing - Page 5

**Supplementary Figure 1 Zone-wise distribution of rotavirus positivity and vaccine coverage in India, 2016–2023**

**Supplementary Table 1. Sentinel sites and duration of diarrheal surveillance in India (2016 - 2023)**

| **State (Zone)** | **Date of vaccine introduction**  **& Vaccine name** | **Sentinel Hospital** | **Surveillance period** |
| --- | --- | --- | --- |
| Andhra Pradesh (South) | 20/04/2016  Rotavac | Kurnool Medical College | 04/2016 to 05/2019 |
|  |  | Government General Hospital Kakinada | 08/2017 to 07/2019 |
|  |  | King George Hospital | 05/2016 to 01/2020 |
|  |  | Sri Venkateswara Medical College | 04/2016 to 06/2022 |
| Assam (North-East) | 14/06/2016  Rotavac | Government Medical College Guwahati | 12/2017 to 11/2019 |
|  |  | Baptist Christian Hospital | 04/2018 to 01/2020 |
| Haryana (North) | 11/04/2016  Rotavac | Pandit Bhagwat Dayal Sharma Post Graduate Institute of Medical Sciences | 07/2016 to 12/2023 |
|  |  | Shaheed Hasan Khan Mewati Government Medical College | 04/2016 to 01/2020 |
|  |  | BPS Government Medical College for Women | 05/2016 to 05/2019 |
|  |  | Post Graduate Institute of Medical Education and Research | 09/2016 to 08/2012 |
| Himachal Pradesh (North) | 15/03/2016  Rotavac | Rajendra Prasad Government Medical College | 07/2016 to 03/2020 |
|  |  | Indira Gandhi Government Medical College | 05/2016 to 05/2019 |
| Madhya Pradesh (Central) | 02/04/2017  Rotavac | Mahatma Gandhi Memorial Medical College | 09/2017 to 12/2019 |
| Odisha (East)^*^ | 26/03/2016  Rotavac | Hi-Tech Hospital | 04/2016 to 12/2023 |
|  |  | Sardar Vallabhai Patel Post Graduate Institute of Pediatrics | 03/2016 to 06/2019 |
|  |  | Institute Of Medical Sciences and Sum Hospital | 04/2016 to 06/2019 |
|  |  | Kalinga Institute of Medical Sciences | 04/2016 to 12/2022 |
| Rajasthan (West) | 23/03/2017  Rotavac | Sawai Man Singh Medical College, | 09/2017 to 01/2020 |
|  |  | Dr. Sampurnanand Medical College | 08/2017 to 01/2020 |
|  |  | Rabindranath Tagore Medical College | 08/2017 to 01/2020 |

| Tamil Nadu (South)^*^ | 20/09/2017  Rotavac | Christian Medical College Vellore | 09/2017 to 08/2023 |
| --- | --- | --- | --- |
|  |  | GVMC, Adumkamparai | 10/2017 to 09/2019 |
|  |  | Narayani Hospital and Research Centre | 01/2018 to 08/2019 |
|  |  | Nalam Hospital | 09/2017 to 12/2023 |
|  |  | The Institute of Child Health and Hospital for Children | 09/2017 to 01/2020 and  03/2023 to 12/2023 |
|  |  | Kanchi Kama Koti Child Trust Hospital | 09/2017 to 01/2020 |
|  |  | Government Medical College Madurai | 12/2017 to 01/2020 |
| Uttar Pradesh (North) | 13/08/2018  Rotavac | King George Medical College | 08/2018 to 01/2020 |
|  |  | Institute Of Medical Sciences, Banaras Hindu University | 12/2018 to 12/2019 |
|  |  | BRD Medical College | 08/2018 to 01/2020 |
|  |  | Mangala Hospital and Research Centre | 08/2018 to 12/2019 |
| Gujarat (West) | 01/08/2019  Rotasiil | M P Shah Government Medical College | 11/2020 to 12/2023 |
|  |  | Pandit Deendayal University Medical College and Hospital | 2/2021 to 12/2023 |
|  |  | Surat Municipal Institute of Medical Education & Research | 11/2022 to 12/2023 |
|  |  | B.J.Medical College and Civil Hospital | 12/2022 to 12/2023 |
| Jharkhand (East) | 07/04/2018  Rotasiil | Rajendra Institute of Medical Sciences | 07/2019 to 12/2023 |
|  |  | Rani Hospital | 07/2019 to 12/2023 |
|  |  | Bokaro General Hospital | 07/2019 to 08/2021 |
|  |  | Krishna Murari Memorial Hospital & Research | 09/2021 to 08/2021 |
|  |  | Kshitij Hospital | 12/2019 to 12/2023 |
|  |  | Nidaan Hospital | 02/2020 to 08/2021 |
|  |  | Sadar Hospital | 01/2020 to 03/2020 |
|  |  | PMCH | 12/2019 to 12/2023 |
|  |  | Balajee Hospital | 12/2019 to 12/2023 |
|  |  | Nichitpur Hospital | 12/2019 to 8/2021 |
|  |  | Rani Children Hospital | 12/2019 to 8/2023 |
|  |  | Balpan Hospital | 01/2020 to 7/2021 |
| Karnataka (South) | 26/08/2019  Rotasiil | JSS Medical College and Hospital | 02/2021 to 12/2023 |
|  |  | Kasturba Medical College | 02/2021 to 01/2022 |
|  |  | KLE Prabhakar Kore Hospital | 03/2021 to 09/2021 |
|  |  | KIMS Hubballi | 03/2023 to 12/2023 |
| Kerala (South) | 06/09/2019  Rotasiil | Government Medical College Kozhikode | 07/2021 to 12/2023 |
|  |  | Government Medical College Thiruvananthapuram | 02/2021 to 09/2022 |
|  |  | Jubilee Mission Medical College and Research Institute | 11/2020 to 12/2023 |
| Maharashtra (West) | 20/08/2019  Rotasiil | Lokmanya Tilak Municipal General Hospital | 07/2021 to 02/2022 |
|  |  | MGM Medical College and Research Centre | 12/2020 to 12/2023 |
|  |  | Sant Dnyaneshwar Medical Education and Research Centre | 08/2022 to 12/2023 |
|  |  | GMC Nanded | 02/2023 to 12/2023 |

^*^ Note: In Tamil Nadu and Odisha, the Rotavac vaccine was replaced with Rotasiil in UIP starting in June 2021

**Supplementary Table. 2**  **Distribution of rotavirus positive samples by zone, based on EIA testing**

| **Zones** | **North** | | **South** | | **West** | | **North-East** | | **East** | | **Central** | | **Total** | |
| --- | --- | --- | --- | --- | --- | --- | --- | --- | --- | --- | --- | --- | --- | --- |
| **Study Period** | Samples tested | RVA EIA positive | Samples tested | RVA EIA positive | Samples tested | RVA EIA positive | Samples tested | RVA EIA positive | Samples tested | RVA EIA positive | Samples tested | RVA EIA positive | Samples tested | RVA EIA positive |
| **2016** | 701 | 169 | 523 | 136 | - | - | - | - | 1080 | 425 | - | - | 2304 | 730 |
| **2017** | 1159 | 196 | 994 | 253 | 507 | 150 | 105 | 34 | 1433 | 418 | 2 | 1 | 4200 | 1052 |
| **2018** | 1938 | 372 | 2304 | 409 | 1387 | 270 | 326 | 52 | 1462 | 502 | 179 | 55 | 7596 | 1660 |
| **2019** | 2097 | 397 | 1970 | 334 | 1168 | 188 | 358 | 63 | 952 | 345 | 281 | 114 | 6826 | 1441 |
| **2020** | 213 | 41 | 276 | 47 | 80 | 32 | - | - | 653 | 319 | 1 | - | 1223 | 439 |
| **2021** | 113 | 11 | 471 | 81 | 538 | 120 | - | - | 934 | 425 | - | - | 2056 | 637 |
| **2022** | 138 | 19 | 516 | 104 | 375 | 102 | - | - | 325 | 119 | - | - | 1354 | 344 |
| **2023** | 180 | 21 | 666 | 110 | 708 | 135 | - | - | 749 | 186 |  |  | 2303 | 452 |
| **Total** | 6539 | 1226 | 7720 | 1474 | 4763 | 997 | 789 | 149 | 7588 | 2739 | 463 | 170 | 27862 | 6755 |

**Supplementary Figure 1 Zone-wise distribution of rotavirus positivity and vaccine coverage in India, 2016–2023**


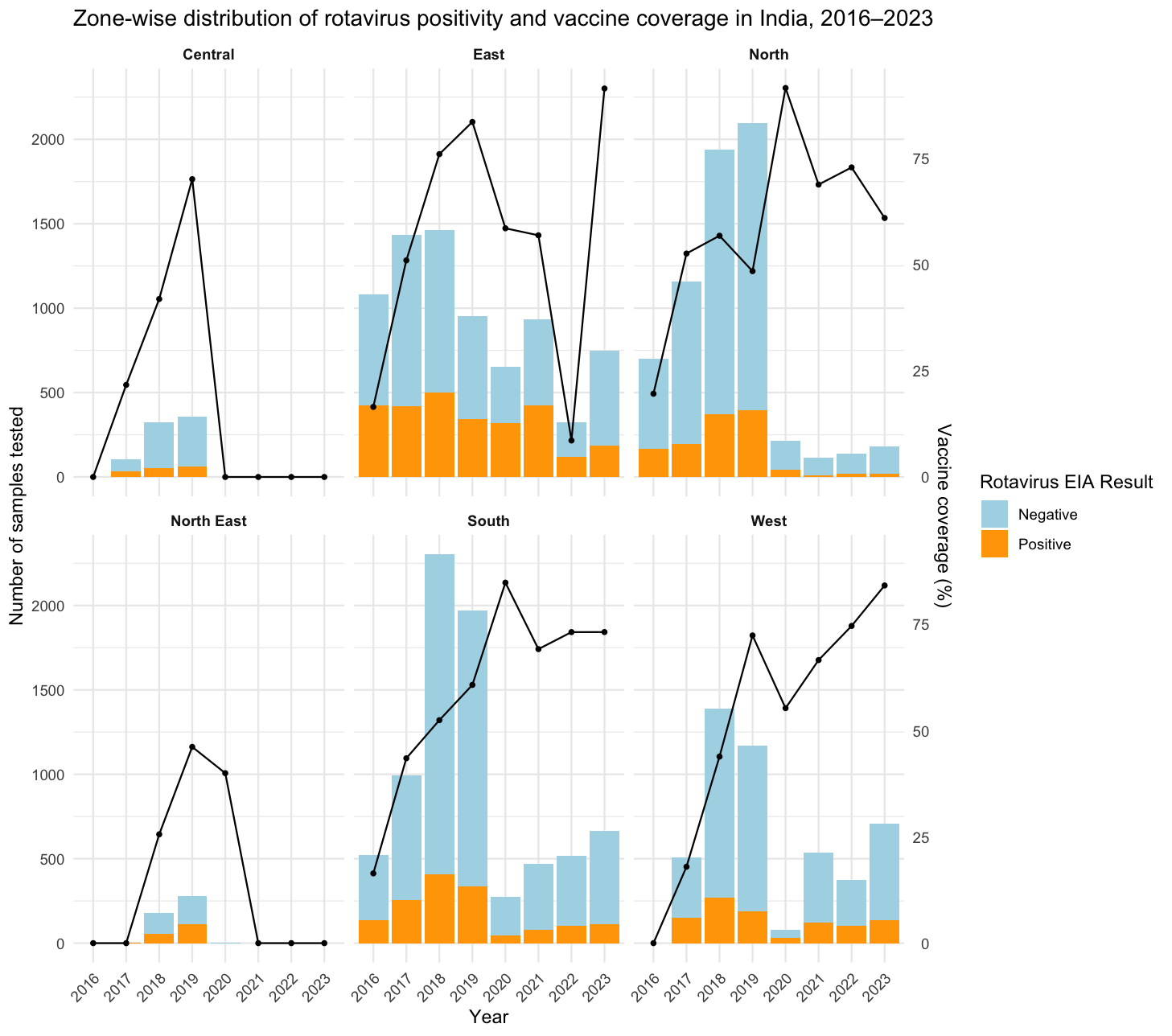

Supplement: Supplementary file 1 — Supplementary Material 1. [file 12985_2026_3126_MOESM1_ESM.docx]
